# Global Trends in Drought Impacts on Wildlife—A Review

**DOI:** 10.1111/gcb.70752

**Published:** 2026-02-13

**Authors:** Leah E. McTigue, Merijn van den Bosch, Hailey M. Boone, Hanna M. McCaslin, Lilly N. Jones, John M. Mola, Zachary L. Steel

**Affiliations:** ^1^ USDA Forest Service Rocky Mountain Research Station Fort Collins Colorado USA; ^2^ Warner College of Natural Resources Forest and Rangeland Stewardship, Colorado State University Fort Collins Colorado USA; ^3^ Graduate Degree Program in Ecology Colorado State University Fort Collins Colorado USA

**Keywords:** amphibians, birds, climate change, drought, mammals, reptiles, wildlife

## Abstract

Drought is expected to increase in frequency and severity due to climate change, highlighting the urgency of understanding drought impacts on wildlife across geographies and taxonomic groups. We conducted a review of peer‐reviewed articles from 1982 to 2024, focusing on the impacts of anomalous drought on terrestrial vertebrates. We recorded 3324 total wildlife responses, 188 from single‐species studies and 3136 from multi‐species studies. Within single‐species studies, 66% of responses were negative, 32% were unclear, and 2% were positive, illustrating the widespread threat of increasing drought to global wildlife. Within multi‐species studies, 24% of responses were negative, and 5% were positive. Notably, 71% of responses within multi‐species papers were categorized as “unclear”, highlighting the need for additional investigation and the complexity of synthesizing a diverse literature. Drought impacts are not evenly tested across taxa, with birds being the most frequently studied (51% of documented responses), followed by mammals (28%), amphibians (16%), and reptiles (5%). Geographically, studies tended to occur most often where recent increases in anomalous drought have been observed (e.g., the Southwestern United States, South Africa, and Southeastern Australia). The sophistication of how drought is measured has increased over time, whereby studies increasingly defined drought as an anomalous event in comparison to a long‐term average through the use of drought indices, rather than as a short‐term weather event. However, we did not see consistency in indices used across the literature, which has the potential to present challenges for interpretation and synthesis. This review summarizes the predominantly negative impacts of drought on terrestrial vertebrates and the growing challenge of conserving wildlife in a changing world, while also highlighting gaps in our understanding of drought‐wildlife relationships that can guide future research.

## Graphical abstract

We reviewed peer reviewed literature pertaining to instances of anomalous drought (any drought event that deviates from standard conditions (e.g., dry season) when compared to a long term average) and its impacts on wildlife. We summarize the directional effects of drought on wildlife, as well as how drought is quantified across the literature.

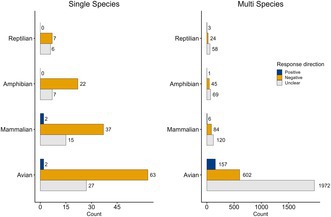

## 1 Introduction

Climate change is predicted to cause widespread declines in biodiversity through direct physiological stress from changing temperature and precipitation regimes, and indirect effects such as habitat deterioration, increased invasive species impacts, and increased pathogen transmission (Thomas et al. 2004; Cahill et al. 2013; Sattar et al. 2021). Drought, although a natural phenomenon, is increasing as the planet warms (Dai 2013; Cook et al. 2020), and there has been an observed global increase in drying conditions over several decades (Chiang et al. 2021; Vicente‐Serrano et al. 2020). Certain areas across the globe, particularly western North America, southern Africa, the Amazon, and Europe, are predicted to see a large increase in the likelihood of extreme drought events (Cook et al. 2020; van den Bosch et al. 2025). These increases are of particular concern for wildlife species sensitive to drought conditions. Reviews assessing drought impact on ecosystems and the impact of climate change on wildlife have been conducted. For example, Chandanpurkar et al. (2025) documented declines in terrestrial water storage, but do not address directly how this will impact wildlife; Vicente‐Serrano et al. (2020) review drought effects on environmental systems, but do not specifically look at drought impacts on wildlife; Pandit and Sharma (2024) focus on the link between climate change and drought, but again are not looking at wildlife impacts; Maxwell et al. (2019) and LeDee et al. (2021) both conduct comprehensive reviews on wildlife, but Maxwell et al. (2019) includes a multitude of extreme weather and climate events and focuses on taxonomic group rather than species level response, and LeDee et al. (2021) is focused on how conservation managers can and are responding to the threat, rather than reviewing drought impacts as we do here. To our knowledge, a comprehensive global review of drought effects on vertebrate wildlife, as well as how drought impacts on wildlife have been quantified, is currently lacking.

Drought can have varied, though often negative impacts on terrestrial wildlife populations (Seabrook et al. 2011; Purcell et al. 2017; Prugh et al. 2018). The magnitude and direction of drought effects likely vary among taxa due to disparate life and evolutionary histories. For example, due to strong associations with waterbodies, amphibian populations may be disproportionately negatively impacted by anomalous drought (Pechmann et al. 1991; Brooks 2009; Walls et al. 2013). Conversely, many reptiles of arid regions may be adapted to periodic drought, but sensitive to directional ecosystem change due to long‐term aridification (Bradshaw 1988; Cox and Cox 2015). Rainfall deficit can directly affect the biology of individuals, which can lead to population‐level changes. For example, dehydration has detrimental effects on the physiological systems (particularly the organs and tissues) of amphibians, decreasing survival (Hillman et al. 2008; Cayuela et al. 2016), and chronic water deprivation in birds and mammals can result in lower body weight (which indicates nutritional stress) to the point of starvation (Fair and Whitaker 2008; Okello et al. 2016; Rey et al. 2017).

Because neither projected changes in drought nor biodiversity are uniform across the globe, drought vulnerabilities are likely to vary geographically. For example, within the continental United States, terrestrial wildlife are predicted to experience an average 377% increase in the frequency of annual drought and an average 579% increase in prolonged (three‐year) drought exposure (Van Den Bosch et al. 2024). Ecoregions such as the Southwest USA that have high predicted increases in drought frequency also have high vertebrate richness and high numbers of species that are considered drought‐threatened by the International Union for Conservation of Nature (IUCN; Jenkins et al. 2013, Van Den Bosch et al. 2024).

Drought has been defined and quantified in many ways, challenging direct comparisons among studies. Drought is often defined as a persistent shortage of precipitation over time, relative to historic measurements (González and Valdés 2006; Quiring 2009). When used properly, drought indices can define drought as anomalous departures from long‐term averages, with over one hundred indices proposed (Zargar et al. 2011). Such indices often convey important aspects of drought beyond precipitation quantities. For example, the Palmer Drought Severity Index (PDSI) provides a “cumulative departure of moisture supply”, and operates independently of season (Palmer 1965; Quiring 2009; Zargar et al. 2011). The Standard Precipitation Index (SPI) provides the difference in actual precipitation from long‐term averages for a specific geographic area, allowing comparisons to be made across space and time (Edwards and McKee 1997). Additionally, drought can be defined as a non‐anomalous event, such as recurring seasonal drought or drought caused by El Niño‐Southern Oscillations (ENSO), which may or may not deviate from long‐term average aridity. Although the impacts of annual dry seasons (e.g., summers in Mediterranean‐type climates) impact wildlife (Donnelly et al. 2018; Vogrinc et al. 2018), projected climatic change underscores the importance of assessing the effects of anomalous drought.

To address the current state of knowledge on the effects of anomalous drought on wildlife, we aimed to answer the following questions: (1) What are the trends in drought definitions and quantifications within vertebrate research? (2) Are documented drought impacts (on vertebrate abundance, demography, body condition, etc.) directionally consistent within and among taxonomic groups? (3) What are the gaps in research pertaining to taxonomic groups and geographic locations, and how do these align with observed spatial patterns in changing drought frequencies? By answering these questions, we aim to provide a foundation for identifying methodological commonalities or inconsistencies, knowledge gaps, and patterns within wildlife responses to increases in drought frequency and intensity.

## 2 Methods

To assess the current body of literature, we systematically compiled and reviewed literature on anomalous drought impacts on wildlife communities. We define anomalous drought as a period of uncharacteristically dry conditions in a region as compared to a long‐term average. Wildlife considered include non‐domestic terrestrial vertebrates (i.e., excluding livestock, fish, and invertebrates). We included amphibians due to many species having terrestrial life stages and their overall sensitivity to drought events. We searched the Web of Science database (2025 Clarivate) with no filter placed on the date published. We set the query to search the Topic (TS), which includes the title, abstract, and keywords in the search:

TS = (Drought AND wildlife) OR (Drought AND mammal) OR (Drought AND reptile) OR (Drought AND amphibian) OR (Drought AND bird) OR (Drought AND avian) OR (Drought AND bat) OR (Drought AND herp*)

This initial search yielded 2332 articles. From here, we conducted three rounds of review on the collected literature. For Round 1, we manually reviewed each title and abstract and retained studies that discussed drought impacts on non‐domestic terrestrial vertebrates. We opted not to “snowball” citations to maintain consistency in study inclusion among multiple reviewers. This resulted in 522 articles incorporated in our review.

In Round 2, we mapped how drought was quantified across the literature. We tallied and excluded articles that did not address anomalous drought studies focusing on seasonal drought, recurring ENSO‐related droughts, and drought events not placed within the context of a long‐term average (e.g., “the dryest year in four years”). We excluded any study representing non‐original data, including synthesis or simulation studies. We recorded drought quantification in various ways: (1) Was there explicit quantification of a drought event (vs. simply noted as having occurred)? (2) Was drought further quantified as anomalous? Drought was considered “quantified anomalous” when empirically compared to long‐term conditions of a period ≥ 30 years. (3) What was the metric used to quantify drought in each study? We chose 30 years as a cutoff for explicit quantification of anomalous drought because this has been identified as sufficient to compare current trends to long‐term conditions (Turner 2008). If drought was quantified as anomalous, we collected data on the description of drought itself, categorizing it into one of three groups: categorical (effect of drought vs. no drought), severity (effect of precipitation, SPI, or other drought metric), and frequency (effect of number of drought occurrences over time).

To address our second research question, for each study, we recorded the direction of the effect of drought on the species under consideration. We classified response direction into three categories: positive (increased survival, fecundity, abundance, food intake, etc.), negative, and unclear. We defined the unclear category as any result where the impact of drought did not clearly influence a species vital rate (e.g., direct mortality, changes in fecundity, body condition, abundance, occupancy). This includes studies that either (1) showed an effect magnitude that was too small to discern directionality given the study's methodology, (2) the sample size or methodology resulted in high uncertainty and no clear estimated effect, and (3) an effect was observed but it was unclear whether there would be positive or negative effects on a population (e.g., shift in habitat use without an accompanying change in fitness). We broke down the studies to obtain species‐level responses by recording the number of species included in each study, and how many of those species were shown to have a positive, negative, or unclear result. In cases where the study did not specify how many species were included in the study, it was treated as a single‐species study, and a single response was recorded. Because multiple studies may assess the same species, our results should be interpreted as a summary of total responses reported in the literature rather than a count of individual species.

We compared the geographic distribution of drought studies that addressed anomalous drought to recent globally observed changes in drought frequency. Observed changes in drought were measured as the percentage of months considered part of an annual drought as defined by the Standardized Precipitation Evapotranspiration Index (SPEI), derived from the SPEI Global Drought Monitor (Beguería et al. 2014). SPEI values represent the deviation of the climatic water balance from a historical average (1950–2010), and the value distribution has a mean of 0 and a standard deviation of 1, with negative SPEI values indicating drier conditions than average. Using a moving time window, SPEI‐12 values represent the cumulative water balance over a given number of months (i.e., 12 months) preceding the target month. For each grid cell and each study location, we calculated the difference in the percentage of months considered part of an annual drought (SPEI‐12 < −1.5) for the 1951–1981 and 1993–2023 periods at a resolution of 0.5°, respectively. Values were bound between −100% and 100%, whereby 100% implied a change from no annual drought during the first period to continuous annual drought during the second period.

Finally, we calculated the percentage of global land area where annual drought increased versus the percentage of study locations where it increased. Since some studies encompassed extremely broad geographies (e.g., eastern United States or Kenya), we derived the comparison from a subset of our original data with sub‐regional location data (*n* = 189). We included locations for studies that met at least one of the following criteria: (1) The study provided specific GPS coordinates for their study site. (2) The study mentioned a particular wildlife refuge, town, or army base for which we could derive a GPS location (e.g., Mason Mountain WMA, TX; Fort Hood, TX). (3) The study took place within a specific geographic location with relatively consistent habitat and weather patterns (e.g., Kalahari Desert, Warrumbungle Mountains), excluding large areas such as states or countries.

Each qualifying study was reviewed by a primary reviewer as described above, followed by a quality assurance check by a different secondary reviewer to ensure that data were accurately extracted and reviewer bias minimized. Any discrepancies between the data pulled by each reviewer were reconciled by L. McTigue and M. van den Bosch. All data analysis and visualization of the data (McTigue et al. 2026) was conducted in R Studio (4.4.2, RStudio: Integrated Development for R. RStudio, PBC, Boston, MA).

## 3 Results

Out of the 522 studies under consideration, 278 (53%) assessed instances of anomalous drought effects on terrestrial wildlife. Study publication dates ranged from 1982 to 2024. Of the 278 studies addressing anomalous drought, drought was explicitly quantified using the amount of precipitation, a standardized drought index, or similar metric in 227 studies (81%), and of these, 152 studies explicitly quantified the observed drought event as anomalous (54%). Only 40% of the reviewed studies hypothesized a likely mechanism of drought effects (e.g., dehydration of individuals or eggs, drought‐caused food shortage, etc.), but very rarely was it empirically tested (3% of studies were experimental).

Within the 278 studies included in our final dataset, 170 studies had only one focal species, while the others were multi‐species research. The most species studied within a single study was 445 (Albright et al. 2010b). We recorded 2769 avian responses, 264 mammalian responses, 144 amphibian responses, and 68 reptilian responses.

**FIGURE 1:**
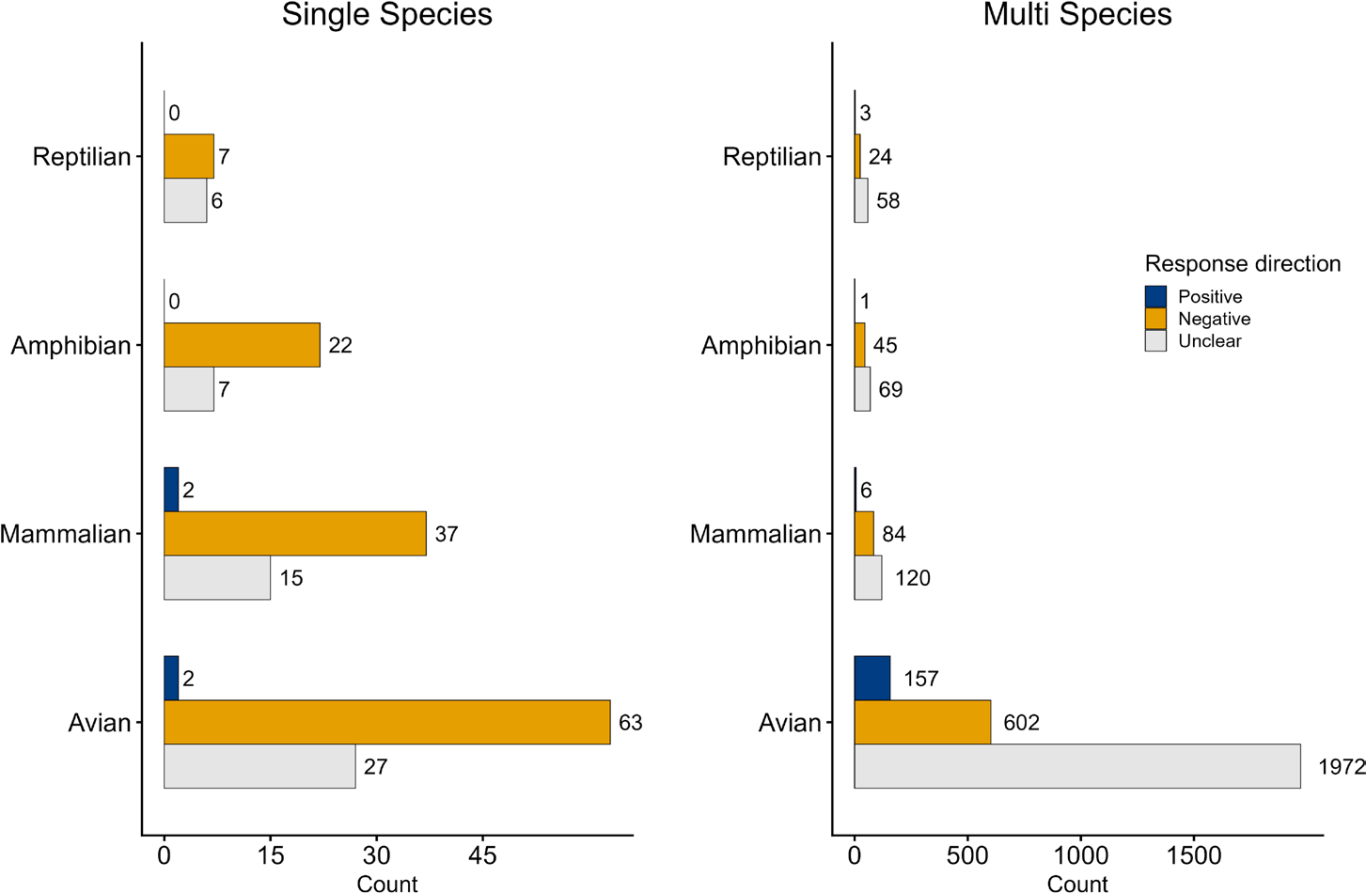
Summary of species response by taxa. The positive responses tally documented benefits from drought (e.g., increase in survival, fecundity, etc.). Negative responses refer to instances of observed harm to a species (e.g., decreased survival, fecundity, etc.) The unclear category included instances of insufficient evidence of a directional effect (see methods for full definition).

Within single‐species papers, avian response was positive 2% of the time (*n* = 2), negative 68% of the time (*n* = 63), and unclear 29% of the time (*n* = 27). Mammal responses within single‐species papers were 4% positive (*n* = 2), 69% negative (*n* = 37), and 28% unclear (*n* = 15). Amphibian responses were 0% positive (*n* = 0), 76% negative (*n* = 22), and 24% unclear (*n* = 7). Reptiles were 0% positive (*n* = 0), 54% negative (*n* = 7), and 46% unclear (*n* = 6) (Figure 1).

Within multi‐species papers, avian response was positive 6% of the time (*n* = 157), negative 22% of the time (*n* = 602), and unclear 72% of the time (*n* = 1927). Mammal responses within multi‐species studies were 3% positive (*n* = 6), 40% negative (*n* = 37), and 57% unclear (*n* = 120). Amphibian responses for multi‐species studies were < 1% positive (*n* = 1), 39% negative (*n* = 45), and 60% unclear (*n* = 69). Reptiles were 4% positive (*n* = 3), 28% negative (*n* = 24), and 68% unclear (*n* = 58) (Figure 1).

**FIGURE 2:**
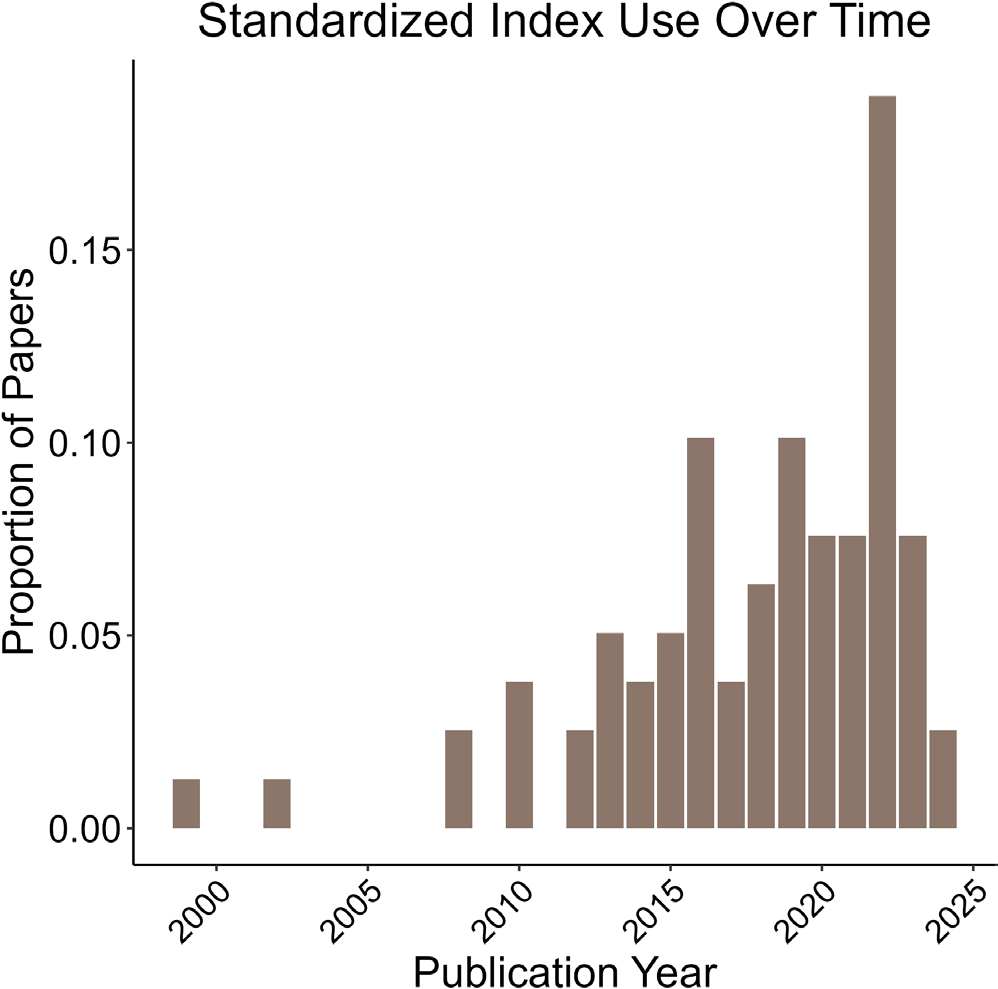
The proportion of standardized drought indices used in wildlife studies over time. Use of standardized indices ranges from 1999 to 2024. Bars represent the proportion of all reviewed studies that used drought indices (*n* = 171).

Studies included in our review were nearly all observational (*n* = 263), with few experimental studies (*n* = 8), and three that did not fall into these categories and were labeled as “other”. Precipitation was the most common drought metric used (47% of studies), though the use of drought indices increased over time (Figure 2) and indices were used in 78 of the 278 (28%) studies.

**FIGURE 3:**
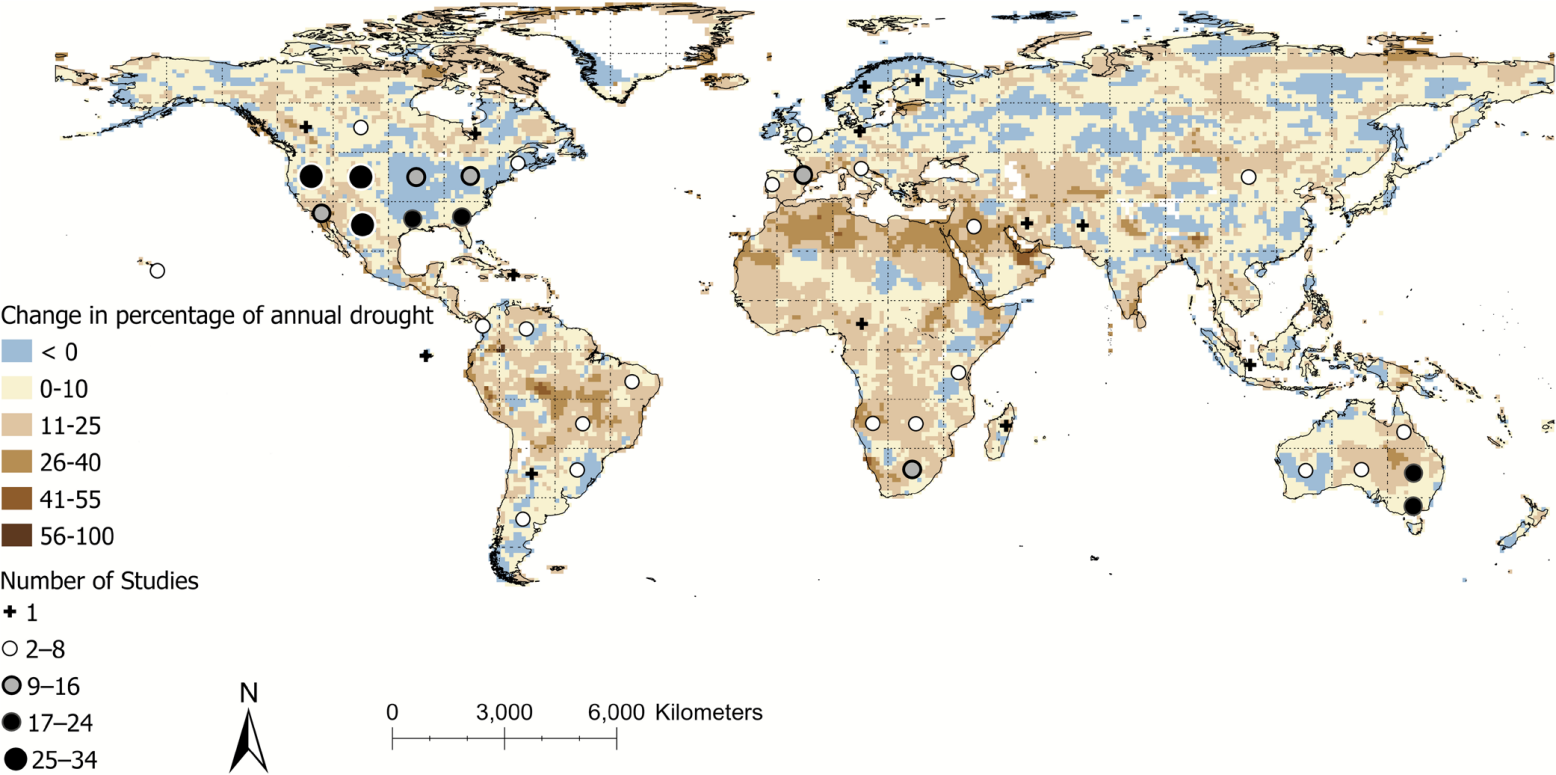
Global distribution of drought studies (*n* = 278) from 1982 to 2024, along with observed changes in the percentage of months considered part of an annual drought (SPEI‐12) between 1951–1981 and 1993–2023. Study locations are grouped within a ~11° grid for visualization purposes and do not represent the exact location of any particular study.

Studies involving anomalous drought came from 6 continents. North America was the most frequently studied continent (*n* = 164), followed by Australia (*n* = 48), Africa (*n* = 24), Europe (*n* = 19), South America (*n* = 12), Asia (*n* = 6), and one study that involved locations in both Asia and South America (Figure 3).

**FIGURE 4:**
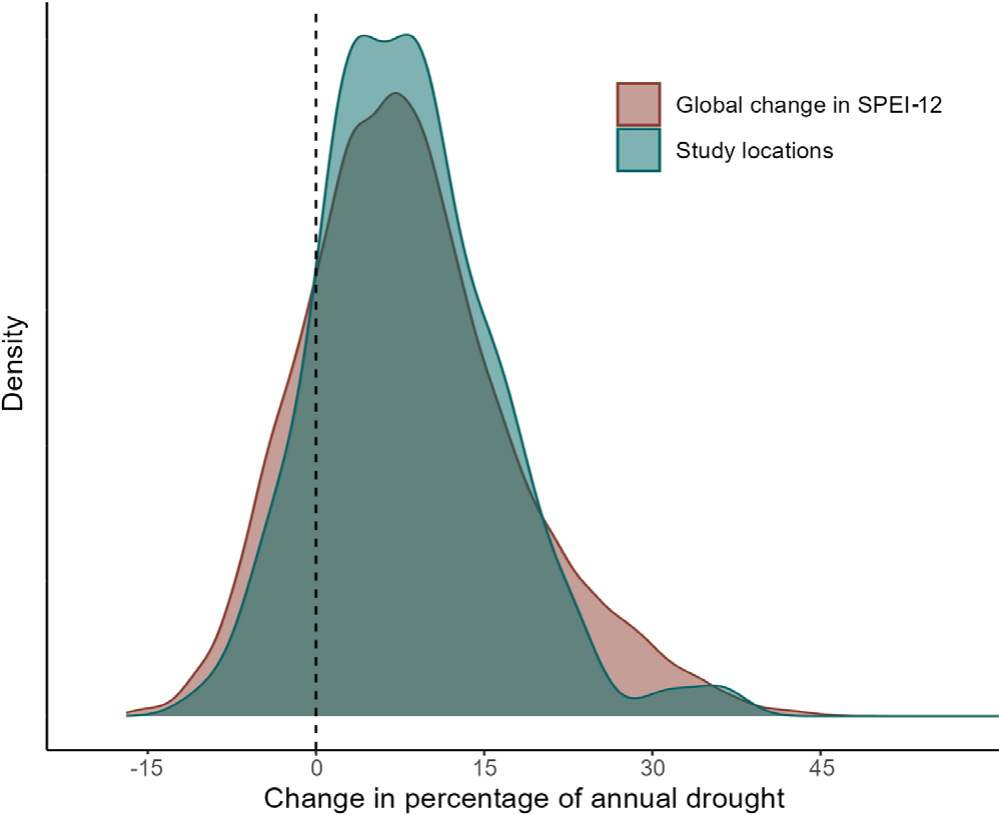
Distribution plot comparing the observed changes in the percentage of months experiencing annual drought (SPEI‐12) between 1951–1981 and 1993–2023 for all study locations, compared to the global distribution of observed changes for the same time periods.

We saw a large overlap in increases of annual drought frequency between the global mean (Mean increase of 8.52%, 95% CI; 8.38%–8.66%) and the study locations (Mean increase of 8.24%, 95% CI; 7.29%–9.18%) included in the distribution analysis (Figure 4). Published studies were primarily conducted in areas with observed increases in drought frequency, indicating these areas have been well‐studied. However, there are noticeable gaps in South America and Northern Africa, including areas with strong increases in drought frequencies (Figures 3 and 4).

## 4 Discussion

Our review compiles drought research over a 42‐year period (1982–2024), spanning six continents and four taxonomic groups of terrestrial vertebrates. Scientific attention to this topic increased between 1982 and 2024, coincident with increased frequency and severity of drought across many areas of the globe. The reviewed studies showed a broad but heterogeneous vulnerability of terrestrial wildlife to increases in anomalous drought. There is a clear bias in the geographic and taxonomic coverage, which illustrates opportunities for continued research in this growing field.

Following decades of drought research (Gorham and Kelly 2018) and a wide array of available drought metrics (Wilhite and Glantz 2009), the increased research attention on the impacts of drought on wildlife is characterized by little consistency in method or metrics of drought employed. Although the field is beginning to adopt techniques enabling a mechanistic understanding of how drought impacts wildlife directly, the proliferation of novel approaches for quantifying drought presents challenges for interpretation and synthesis. Further, the vast majority of studies remain observational, highlighting a common challenge in ecology of linking outcomes to mechanisms. The multitude of drought indices available to researchers provides flexibility, but challenges synthesis and consistent understanding of drought impact. When practicable, we suggest the use of SPEI as it takes both precipitation and evapotranspiration into account (Zargar et al. 2011; Beguería et al. 2014; Slette et al. 2019). Only 52% of our initial search in the Web of Science (2025 Clarivate) pertained to drought as an anomalous phenomenon, indicating many studies had more opportunistic definitions of drought whereby weather conditions during the study period may not actually have deviated from long‐term climate conditions (e.g., “the two driest years of the decade” are not necessarily anomalously dry years). Within our reviewed studies, many different definitions of drought were used, with 11% (*n* = 31) not explicitly quantifying drought at all. Additionally, 91 studies (33%) quantified drought, but did not specifically quantify it as anomalous (drought conditions compared to a long‐term average). Overall, despite widespread indications that anomalous droughts will increasingly affect vertebrate populations, less than a third of studies have attempted to quantify anomalous drought effects on vertebrates.

Drought resulted in negative outcomes for terrestrial vertebrates in over half of single‐species responses (68%, *n* = 129) and a quarter of multi‐species responses (*n* = 755). Among the literature describing negative outcomes for vertebrates, studies were primarily focused on mammalian and avian species (*n* = 108), with herpetofauna remaining relatively understudied (27 studies focused on amphibians and 6 on reptiles). Mechanisms were rarely tested directly, but authors hypothesize a range of causes including decreased nesting success due to food scarcity of southern grey shrikes ( *Lanius meridionalis* ) in Israel (Keynan and Yosef 2010), decreased body temperatures followed by death due to lack of prey availability and negative energy balance in aardvarks ( *Orycteropus afer* ) in the Kalahari desert of South Africa (Rey et al. 2017), and limited breeding success in gopher frogs ( *Rana capito* ) in the southeastern United States, linked to decreased rainfall and reduction in ephemeral wetlands (Crawford et al. 2022). The uneven taxonomic coverage is especially problematic as herpetofauna are likely to be particularly vulnerable to drought (Araújo et al. 2006; Twomey et al. 2025). For instance, drought can increase the prevalence of invasive species such as bullfrogs, displacing native amphibians, decreasing their overall density, or increase the spread of chytrid fungus (*Batrachochytrium dendrobatidis*), which has devastated amphibian populations across the globe (Adams et al. 2017).

We found 171 instances (5%) of positive responses to drought across all studies. This was primarily seen within multi‐species avian studies, where positive responses sometimes coincided with a much larger number of negative responses. One example of a positive response in birds is an observed increase in eggshell thickness for mountain plovers ( *Charadrius montanus* ) under drought conditions, which may support greater reproductive success (Dreitz et al. 2012). Some mammals (*n* = 8) also displayed positive responses to drought, including an increased abundance of the southern grasshopper mouse ( *Onychomys torridus* ) during drought conditions (Prugh et al. 2018). We saw a positive response to drought in amphibians only once. Davis et al. (2017) describe how in periods of drought, the ornate chorus frog ( *Pseudacris ornata* ), which breeds exclusively in ephemeral wetlands, experiences less predation by fish that are unable to persist under drought.

Unclear responses were the largest category among multi‐species studies and illustrate challenges to synthesizing the current literature. The unclear category includes all responses that could not be clearly defined as positive or negative (e.g., change in space use or diet), showed no significant response to drought, or were studies that looked at the effects of drought but did not disclose species‐by‐species effects. A large proportion of unclear responses were derived from articles where the number of species under consideration was disclosed, but drought effects were reported on the community‐wide or guild level (e.g., Albright et al. 2010a, 2010b). Non‐significance can be attributed to either small effect sizes and/or insufficient statistical power to discern a clear directional impact. For example, one study documented how tule elk (*Cervus canadensis nannode*) shifted its foraging location to be closer to water in drought conditions (Mohr et al. 2022), while other studies provided evidence of changes in diet in response to drought (Gedir et al. 2016; Smith et al. 2022), but with no corresponding likely effect on the populations. Research linking these shifts in space use or behavior to demographic outcomes would bolster our overall understanding of how drought impacts wildlife and is a clear avenue for future research.

Our review included only articles published in the English language, which may have contributed to the uneven geographic representation of drought and wildlife studies. For example, the Middle East and Southeast Asia could be underrepresented in our review, both due to different publication languages and ongoing conflict in these regions, which creates constrained research funding (Khattak 2009; Nussbaumer‐Streit et al. 2020). However, we expect this comprehensive review of English language studies is representative of the broader literature with respect to the general impacts of drought among well‐studied taxa (Nussbaumer‐Streit et al. 2020).

There is high overlap between geographic locations that have experienced past increases in drought and locations where drought‐related vertebrate research has been conducted (Figure 4). This suggests past research represents the distribution of contemporary drought, albeit with notable geographic and taxonomic biases. Areas such as the US and South Africa have been relatively well studied, though we see research gaps in areas that are projected to see large changes in drought severity, including Northern Africa (particularly the northern coasts of Morocco, Algeria and Tunisia), Australia's western coast (between Perth and Albany), and South America (predominantly the coasts of Venezuela and Southern Chile, and southern parts of the Amazon Basin) (Figure 3). Drought intensity is predicted to increase in regions such as the Amazon Basin, Central America, the southwestern coast of South America, North and South Africa, as well as China and Australia (Duffy et al. 2015; Naumann et al. 2018). Many of these locations, especially those centered along the equator, also hold the highest biodiversity in the world, particularly for terrestrial species (Bowker 2000; Jenkins et al. 2013). Vertebrates in the Amazon Basin may experience significant habitat loss or degradation due to climate change (Trew and Maclean 2021), while also facing increased physiological stress due to severe increases in extreme heat and drought (van den Bosch et al. 2025). Future research would be most useful if focused on understudied taxa as well as specific regions that are predicted to experience the greatest future drought increases under a broad range of climate models.

Reviews such as this one are valuable to capture trends in research and highlight gaps in scientific understanding, whether taxonomic, geographic, or methodological. As drought research becomes increasingly prevalent, highlighting understudied areas will help direct future investigations where it is most needed, bolstering the existing body of literature. Further, we highlight the importance of quantifying drought as an anomalous condition, which is indispensable to further our understanding of drought impacts on wildlife communities under climate change.

## Author Contributions

**Leah E. McTigue:** conceptualization, data curation, formal analysis, investigation, methodology, supervision, writing – original draft, writing – review and editing. **Merijn van den Bosch:** data curation, formal analysis, investigation, visualization, writing – review and editing. **Hailey M. Boone:** investigation, writing – review and editing. **Hanna M. McCaslin:** investigation, writing – review and editing. **Lilly N. Jones:** investigation, writing – review and editing. **John M. Mola:** investigation, supervision, writing – review and editing. **Zachary L. Steel:** conceptualization, investigation, methodology, supervision, writing – review and editing.

## Funding

This work was supported by Rocky Mountain Research Station and the California Department of Fish and Wildlife (Grants 23‐JV‐11221635‐076 and D2380002).

## Conflicts of Interest

The authors declare no conflicts of interest.

## Data Availability

All data used in our review is publicly available and can be found at https://doi.org/10.5061/dryad.nk98sf877.
